# Research Hotspots in Psoriasis: A Bibliometric Study of the Top 100 Most Cited Articles

**DOI:** 10.3390/healthcare11131849

**Published:** 2023-06-26

**Authors:** Oana Mirela Tiucă, Silviu Horia Morariu, Claudia Raluca Mariean, Robert Aurelian Tiucă, Alin Codruț Nicolescu, Ovidiu Simion Cotoi

**Affiliations:** 1 Doctoral School of Medicine and Pharmacy, University of Medicine, Pharmacy, Science, and Technology George Emil Palade of Targu Mures, 540142 Targu Mures, Romania; 2 Dermatology Department, University of Medicine, Pharmacy, Science, and Technology George Emil Palade of Targu Mures, 540142 Targu Mures, Romania; 3 Dermatology Clinic, Mures Clinical County Hospital, 540342 Targu Mures, Romania; 4 Pathophysiology Department, University of Medicine, Pharmacy, Science, and Technology George Emil Palade of Targu Mures, 540142 Targu Mures, Romania; 5 Endocrinology Department, University of Medicine, Pharmacy, Science, and Technology George Emil Palade of Targu Mures, 540142 Targu Mures, Romania; 6 Endocrinology Department, Mures Clinical County Hospital, 540139 Targu Mures, Romania; 7 Agrippa Ionescu Emergency Clinical Hospital, 011773 Bucharest, Romania; 8 Pathology Department, Mures Clinical County Hospital, 540011 Targu Mures, Romania

**Keywords:** psoriasis, therapy, immunopathogenesis, bibliometry, citation impact

## Abstract

(1) Introduction: Psoriasis is a chronic, immune-mediated disease that negatively impacts patients’ quality of life and predisposes them to cardiovascular or metabolic diseases. This paper aims to summarize the knowledge structure and future directions in psoriasis research by means of bibliometrics. (2) Material and methods: The Thomson Reuters Web of Science database was interrogated using preestablished keywords. A list of the top 100 most cited articles focusing solely on psoriasis was compiled and analyzed. VOSviewer software was used to assess and visualize collaboration networks, citation, co-citation and co-wording analysis, and bibliographic coupling. (3) Results: The articles were written by 902 authors from 20 countries and were published in 31 journals. The United States was at the forefront of this field. Griffiths, CEM had the most citations, while the most prolific institution was Rockefeller University, New York City. Pathogenesis, especially key-pathogenic factors, immune pathways, and epidemiology were the most discussed topics. Work published in the last decade focused on the use of biologics. Keywords such as “quality of life”, “efficacy”, and “necrosis-factor alpha” have been widely used. (4) Conclusion: Research interest regarding psoriasis is high, leading to the rapid development of this field. Treatment modalities, especially novel-targeted therapies, immune pathways, and an integrative approach to such cases are receiving great interest and represent research hotspots in the future.

## 1. Introduction

Psoriasis is a chronic, immune-mediated disease that negatively impacts patients’ quality of life (QoL). In the last published global report [1], the World Health Organization reported an increasing prevalence of psoriasis, ranging between 1.5% and 5% in developed countries [2]. Its etiopathogenesis is complex, with genetic predisposition, an altered immune response, and various triggering factors concurring in the development of this disease [3,4]. Clinically defined by cutaneous erythema, scaling, and induration, and in some cases by joint and nail involvement, this disease seems to predispose patients to a higher risk of developing cardiovascular disease, diabetes, dyslipidemia, and metabolic syndrome [5,6].

A continuous stream of research is being conducted in relation to psoriasis, aiming to shed light on the pathogenesis, management, and therapeutic outcomes of this disease. A thorough study of scientific advances in a specific field may lead to improvements in the diagnosis and treatment of various diseases. In order to evaluate progress in psoriasis research and its future directions, a bibliometric analysis is of great use.

This concept was proposed by Pritchard [7] and uses statistical parameters to identify emerging trends and collaboration patterns between research constituents. Citations are the most forthright measure of a paper’s impact [8]. Additionally, performance analysis, which illustrates the contributions of research constituents, and science mapping, which depicts the relationships between them, provide additional insight into the academic significance of research papers.

Nevertheless, the use of bibliometry in medical research is relatively new. It has been sparsely utilized, especially regarding cancers [9,10,11,12,13]. However, to the best of our knowledge, this is the first paper to address overall research directions in psoriasis, taking into account bibliometric algorithms based on the top 100 most cited articles referring to this disease.

## 2. Material and Methods

### 2.1. Search Strategy and Data Collection

The search was conducted on the Thomson Reuters Web of Science (WoS) database on 15 January 2023. The following keywords were used: “psoriasis”, “plaque psoriasis”, “guttate psoriasis”, “erythrodermic psoriasis”, and “pustular psoriasis”, separated by the Boolean OR. Articles from all fields were searched across the entire database without regard to article type or study design. Citations recorded in the indexing database are as follows: Science Citation Index Expanded, Social Sciences Citation Index, Conference Proceeding Citation Index-Social Science and Humanities, Conference Proceedings Citation Index-Science, and Emerging Sources Citation Index.

**Figure 1:**
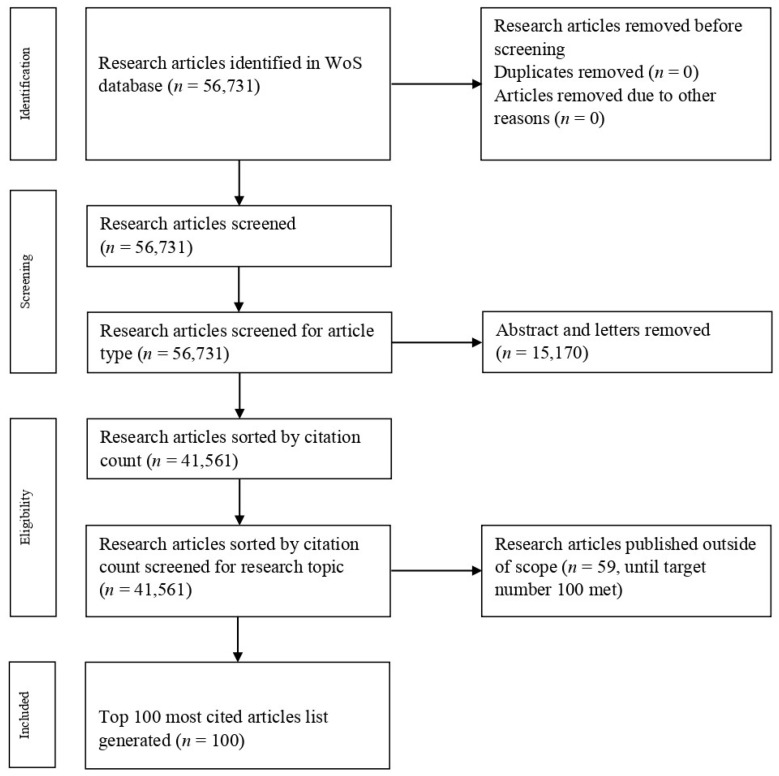
Prisma diagram of workflow.

The search returned 56,731 results, that were afterward screened. Abstracts and letters were excluded. Only full-length English articles were considered. The returned articles were sorted by citation count using Paladugu’s method [14]. Articles focusing on psoriatic arthritis or other inflammatory or autoimmune skin disorders were excluded. Papers referring only to psoriasis-specific topics, such as pathogenesis, treatment modalities, or outcomes, were reviewed. Search and screening of the results were made by two independent researchers to ensure relevance to the selected topic. Disagreements were resolved by discussion between the two involved researchers. The Prisma diagram (Figure 1) exemplifies the workflow. A list of the top 100 most cited articles was compiled and analyzed for various parameters over the next four weeks [15,16,17,18,19,20,21,22,23,24,25,26,27,28,29,30,31,32,33,34,35,36,37,38,39,40,41,42,43,44,45,46,47,48,49,50,51,52,53,54,55,56,57,58,59,60,61,62,63,64,65,66,67,68,69,70,71,72,73,74,75,76,77,78,79,80,81,82,83,84,85,86,87,88,89,90,91,92,93,94,95,96,97,98,99,100,101,102,103,104,105,106,107,108,109,110,111,112,113,114].

### 2.2. Bibliometric Analysis

Information regarding the journal, authorship, institution, publication year, and study design was extracted for the selected articles. Clarivate Journal Citation Reports was used for each journal’s 2021 and 5-year impact factors. Full data regarding the selected articles were generated from the WoS database as an Excel spreadsheet and as a plain text file.

Publication-related metrics were analyzed with Microsoft Excel software. Science mapping and data visualization were performed with VOSviewer software (Version 1.6.19-2023). VOSviewer is a graphical user interface-based free software first developed by van Eck and Waltman in 2010 [115], with the latest version launched on 23 January 2023. It allows the analysis and visualization of different collaboration patterns between research constituents.

The following performance analysis parameters were evaluated: publication-related metrics (total publications, solo-authored publications, co-authored publications) and citation-related metrics (total citations and citation rate). The citation rate was calculated by dividing the total number of citations by the number of years since publication. The bibliometric and knowledge structure of the research field is evaluated in this paper by using the following science mapping techniques: citation, co-citation, co-word, co-authorship analysis, and bibliographical coupling. The counting method was set at full counting. To limit spelling differences in authors’ or institutions’ names an additional thesaurus file, that gives consistent labels to the same word spelled differently, was generated, and used when appropriate.

## 3. Results

### 3.1. Citation Analysis

The total citation count for the analyzed articles was 68,691, with a median of 553.5 and a mean of 686.91. Eighty-four were original articles, while sixteen were reviews. Pathogenesis and epidemiology were the topics most discussed (*n* = 66), followed by management (*n* = 45) and genetics (*n* = 9). Twenty-six articles focused on the use and effectiveness of novel targeted immune therapies, such as biologics, in the management of moderate-to-severe plaque psoriasis. Pustular psoriasis was addressed in two articles [45,63].

Within the top 100, the citation count ranged between 405 for “Clinical response to adalimumab treatment in patients with moderate to severe psoriasis: Double-blind, randomized controlled trial and open-label extension study” by Gordon, K et al. [28] and 2145 for “Severe psoriasis—oral therapy with a new retinoid” by Fredriksson, T et al. [106].

**Figure 2:**
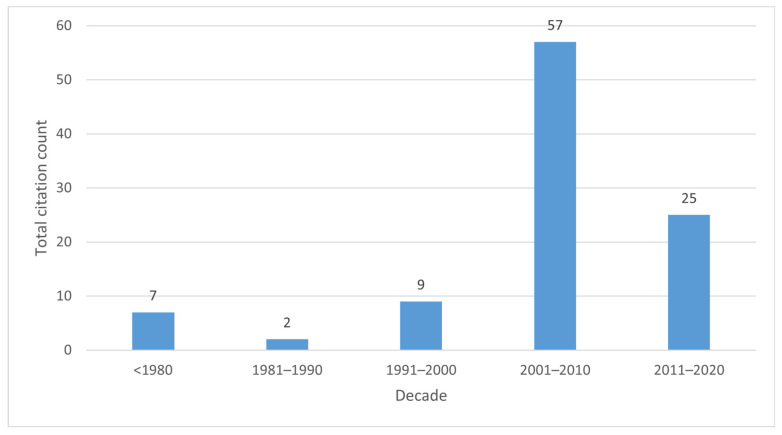
Articles distribution by decade.

The articles were published between 1969 and 2020. The oldest article was “Generalized pustular psoriasis—a clinical and epidemiological study of 104 cases” by Baker, H et al. [45], while the newest one was “Pathophysiology, clinical presentation, and treatment of psoriasis: a review” by Armstrong, AW et al. [73]. They had 421 and 420 citations, respectively. These two articles also have the lowest and highest citation rates, 8 and 211, respectively. Table 1 shows the top 100 articles and their respective citation rates. Figure 2 illustrates the distribution of the articles by decade.

**Figure 3:**
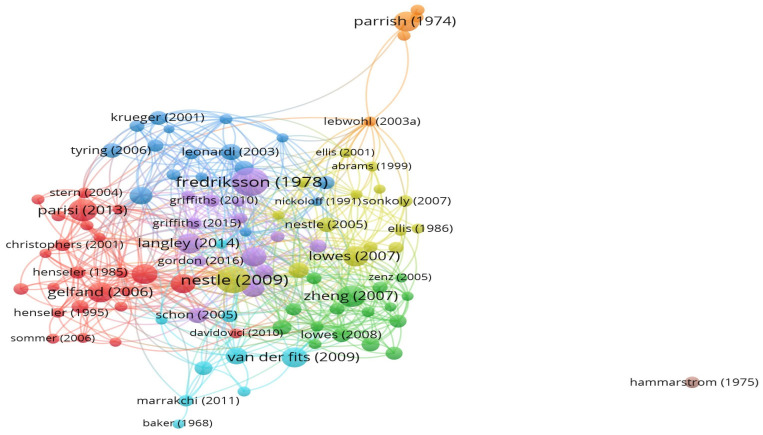
The bibliometric map of the selected articles based on citation patterns. (Cluster colors are as follows: cluster 1—red, cluster 2—green, cluster 3—blue, cluster 4—yellow, cluster 5—purple, cluster 6—turquoise, cluster 7—orange, cluster 8—pink) [15,16,17,18,19,20,21,22,23,24,25,26,27,28,29,30,31,32,33,34,35,36,37,38,39,40,41,42,43,44,45,46,47,48,49,50,51,52,53,54,55,56,57,58,59,60,61,62,63,64,65,66,67,68,69,70,71,72,73,74,75,76,77,78,79,80,81,82,83,84,85,86,87,88,89,90,91,92,93,94,95,96,97,98,99,100,101,102,103,104,105,106,107,108,109,110,111,112,113,114].

Based on bibliometric algorithms that consider citation patterns, such as total citation number and the topic addressed in these papers, the analyzed articles were grouped into eight clusters. Cluster 1 is defined by 24 articles, clusters 2 and 3 by 16 articles each, cluster 4 by 15 articles, cluster 5 by 14 articles, cluster 6 by 9 articles, cluster 7 by 5 articles, and cluster 8 by a single article. The previously mentioned clusters are shown in Figure 3 as map-based connections. Each color represents a thematic cluster, whereas each node represents an author. The size of each individual node and font size is proportional to the number of citations, both related to the completed data set and to each individual cluster.

Griffiths, CEM, Krueger, JG, Papp, K, Krueger, GG, and Menter, A contributed to the greatest number of articles and received a total of 9646, 7669, 8944, 7492, and 6945 citations, respectively. Table 2 highlights the top 10 most cited authors.

The top 100 articles were published in 31 journals, which published between one and sixteen articles. *New England Journal of Medicine* (*n* = 16) published the greatest number of articles within the top 100 and had the highest number of citations (12,817). *Lancet* had the highest impact factor (202.73), published the third-highest number of articles (*n* = 10), and received the third-highest number of total citations (8966). *Dermatologica* had the highest average citation per publication (1301), having published 2 papers with a total of 2602 citations. Table 3 displays articles, citation count, and various journal metrics.

### 3.2. Co-Authorship Analysis

**Figure 4:**
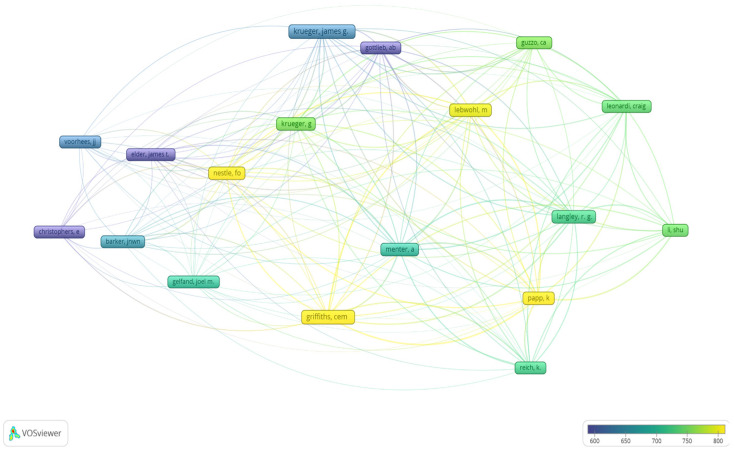
Map-based representation of authors contributing to more than 5 papers scored by average citations. (Color legend: the authors that had the most citations are depicted using yellow frames, while the least cited with purple frames) [15,16,17,18,19,20,21,22,23,24,25,26,27,28,29,30,31,32,33,34,35,36,37,38,39,40,41,42,43,44,45,46,47,48,49,50,51,52,53,54,55,56,57,58,59,60,61,62,63,64,65,66,67,68,69,70,71,72,73,74,75,76,77,78,79,80,81,82,83,84,85,86,87,88,89,90,91,92,93,94,95,96,97,98,99,100,101,102,103,104,105,106,107,108,109,110,111,112,113,114].

The 100 analyzed articles summed 902 authors, out of which 167 contributed to more than 2 papers, while 18 authored more than 5 articles. Three papers were solo-authored [84,86,111]; the highest number of contributing authors for a paper was 136 [49]. Leonardi and Papp first authored the highest number of papers (*n* = 3). Griffiths, CEM (*n* = 12), Krueger, JG (*n* = 12), Papp, K (*n* = 11), Krueger, GG (*n* = 10), and Menter, A (*n* = 10) contributed to the greatest number of articles. The authors who contributed to more than five papers are shown in Figure 4 as map-based connections scored by average citations.

**Figure 5:**
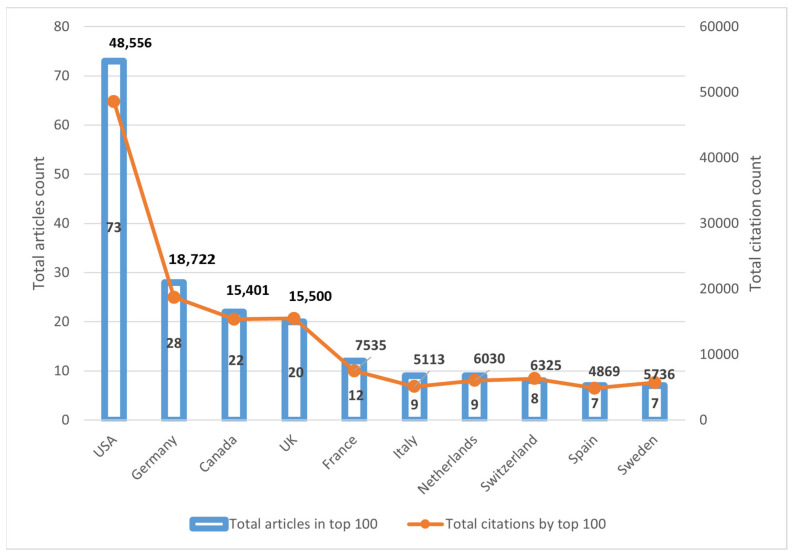
Top countries by total citation count and number of articles.

The authors contributing to the 100 articles originated from 322 institutions and 20 countries. The United States had the most citations (48,556), as well as the highest number of papers (*n* = 73). Germany ranked second, with 18,722 citations from 28 articles. The top five institutions that contributed to the papers were Rockefeller University (*n* = 13), the University of Manchester (*n* = 12), Probity Medical Research (*n* = 11), the University of Michigan, and the University of Utah (*n* = 10, each). Table 4 and Figure 5 depict the top 10 institutions, respectively, countries, that contributed to the top 100 most cited articles.

### 3.3. Co-Word Analysis

**Figure 6:**
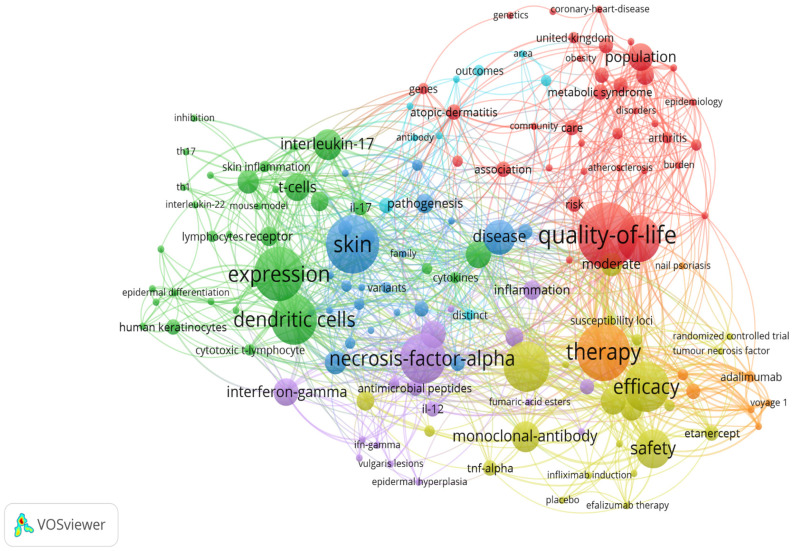
The scientometric map of the keywords from the analyzed articles. (Cluster colors are as follows: cluster 1—red, cluster 2—green, cluster 3—blue, cluster 4—yellow, cluster 5—purple, cluster 6—turquoise, cluster 7—orange) [15,16,17,18,19,20,21,22,23,24,25,26,27,28,29,30,31,32,33,34,35,36,37,38,39,40,41,42,43,44,45,46,47,48,49,50,51,52,53,54,55,56,57,58,59,60,61,62,63,64,65,66,67,68,69,70,71,72,73,74,75,76,77,78,79,80,81,82,83,84,85,86,87,88,89,90,91,92,93,94,95,96,97,98,99,100,101,102,103,104,105,106,107,108,109,110,111,112,113,114].

**Figure 7:**
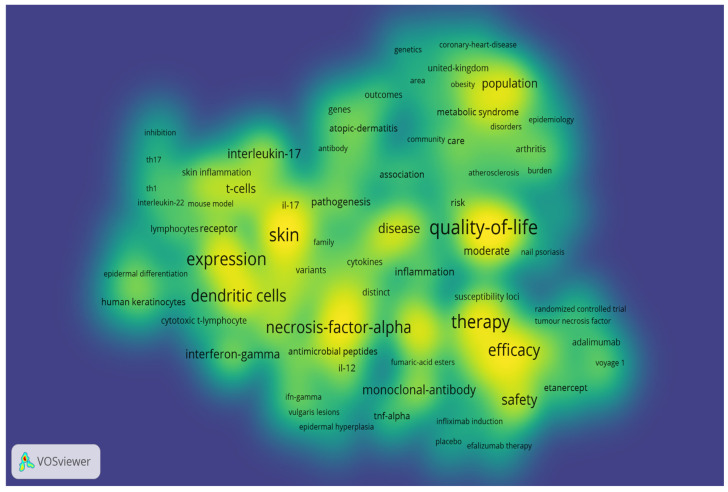
Research tendency based on the density of the keywords used in the 100 articles. (Color legend: the intensity of the yellow and green colors symbolize the frequency of respective keywords).

A total of 471 unique keywords were identified from all articles. After removing keywords such as “psoriasis”, “plaque psoriasis”, “chronic plaque psoriasis”, “severe plaque psoriasis”, “vulgaris”, “to-severe psoriasis”, “psoriasis vulgaris”, and “vulgaris lesions” that could affect the analysis, a minimum threshold of two occurrences was set for each keyword and the bibliometric map was generated. Based on occurrence, they were divided into 7 clusters, as follows: cluster 1 = 37 items, cluster 2 = 28 items, cluster 3 = 23 items, cluster 4 = 19 items, cluster 5 = 18 items, cluster 6 = 12 items, and cluster 7 with 8 items. Figure 6 displays them as map-based connections, while Figure 7 displays keywords occurrence density in the selected articles. A set of the 10 most used keywords was generated and presented in Table 5.

### 3.4. Bibliographical Coupling

**Figure 8:**
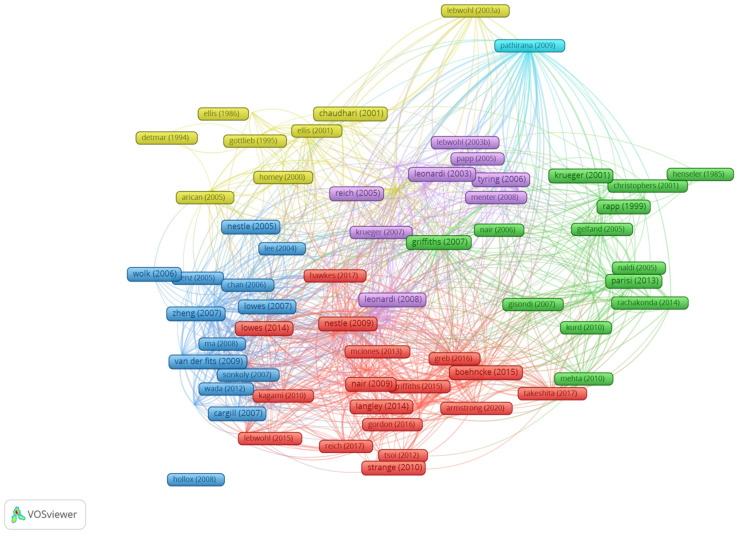
The scientometric map based on existing patterns of citing the same bibliography source. (Due to a high number of bibliographic clusters that led to an overview design when exporting from VOSviewer, this figure depicts the first six clusters, as follows: cluster 1—red, cluster 2—green, cluster 3—blue, cluster 4—yellow, cluster 5—purple, cluster 6—turquoise) [15,16,17,18,19,20,21,22,23,24,25,26,27,28,29,30,31,32,33,34,35,36,37,38,39,40,41,42,43,44,45,46,47,48,49,50,51,52,53,54,56,57,58,59,60,61,62,63,64,65,66,68,69,70,71,72,73,74,75,76,78,79,80,81,82,83,84,85,86,87,88,89,90,91,92,93,94,95,96,97,98,99,100,101,102,103,104,105,107,108,109,110,111,112,113,114].

Based on patterns of citing the same references, the 100 articles were divided into 10 clusters. Cluster 1 consists of 26 items, cluster 2 of 23 items, cluster 3 of 16 items, cluster 4 of 14 items, cluster 5 of 13 items, cluster 6 of 4 items, and clusters 7, 8, 9, and 10 of 1 item each. The map-based connections between all clusters can be found in Figure 8.

### 3.5. Co-Citation Analysis

**Figure 9:**
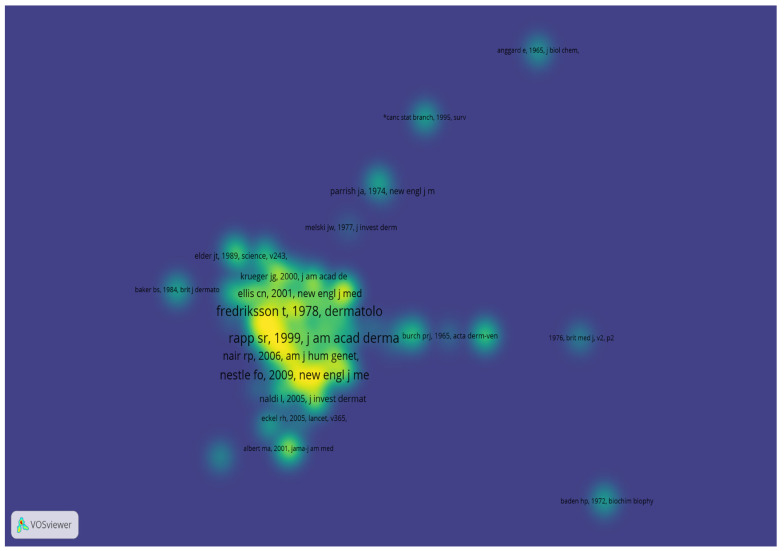
Co-citation tendency. (Color legend: the intensity of the yellow and green colors symbolizes the frequency of respective keywords) [15,16,17,18,19,20,21,22,23,24,25,26,27,28,29,30,31,32,33,34,35,36,37,38,39,40,41,42,43,44,45,46,47,48,49,50,51,52,53,54,55,56,57,58,59,60,61,62,63,64,65,66,67,68,69,70,71,72,73,74,75,76,77,78,79,80,81,82,83,84,85,86,87,88,89,90,91,92,93,94,95,96,97,98,99,100,101,102,103,104,105,106,107,108,109,110,111,112,113,114].

**Figure 10:**
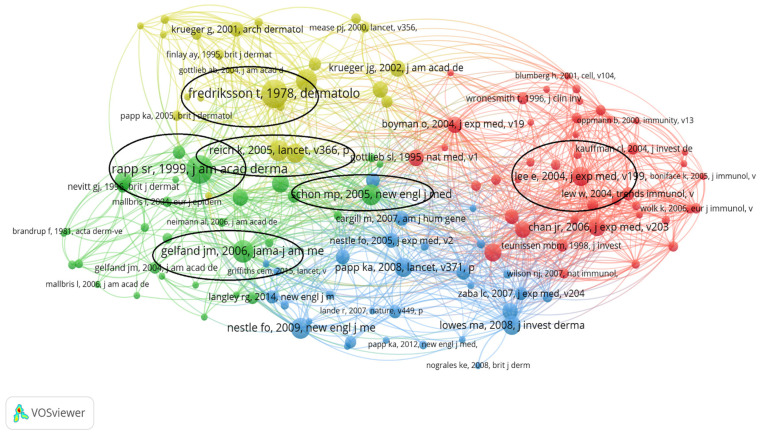
The scientometric map based on co-citation patterns. (Cluster colors are as follows: cluster 1—red, cluster 2—green, cluster 3—blue, cluster 4—yellow) [15,16,17,18,19,20,21,22,23,24,25,26,27,28,29,30,31,32,33,34,35,36,37,38,39,40,41,42,43,44,45,46,47,48,49,50,51,52,53,54,55,56,57,58,59,60,61,62,63,64,65,66,67,68,69,70,71,72,73,74,75,76,77,78,79,80,81,82,83,84,85,86,87,88,89,90,91,92,93,94,95,96,97,98,99,100,101,102,103,104,105,106,107,108,109,110,111,112,113,114].

The analyzed articles summed 3645 references. A minimum threshold of one for each reference was set The most co-cited reference was the article of Rapp, SR et al. [89], which was co-cited by 19 other articles, with 1077 links with other articles and a total link strength of 1358, followed by Fredrickson, T et al. [106], also co-cited by 19 articles, but with lower links (636) and link strength (804), and Gelfand, JM and Leonardi, CL both co-cited 15 times. Figure 9 shows the density of co-cited references. Co-citation frequency is depicted using different intensities of yellow and green. The network of references co-cited more than five times is shown in Figure 10, scored by average citations. They were divided into 4 clusters, as follows: cluster 1 = 43 items, cluster 2 = 30 items, cluster 3 = 30 items, and cluster 4 = 23 items. References located among different clusters and with the highest link strengths are circled with black.

## 4. Discussion

Psoriasis has attracted much interest in the scientific community over the years. In the face of constant evolution and novelties added to the field, it is of great use to maintain the connection to research areas of interest. Bibliometric analysis is able to handle, using quantitative methods, large amounts of literature, to avoid bias usually associated with qualitative-based systematic reviews, and to provide the knowledge structure and future trends of a research topic or field [116].

Among the top 100 articles reviewed, pathogenesis and epidemiology were the topics most often discussed, being the focus of 66 articles. Only 12 articles focused on the clinical and diagnostic aspects of the disease. As psoriasis remains mainly a clinical diagnosis, the emphasis is on better understanding and managing the disease. This points to the fact that the vast majority of the high-impact literature focused on understanding how and why psoriasis develops. This culminated in a trend to explore the role of different immune and inflammatory pathways since the beginning of the 2000s. As key contributors to the immunopathogenesis of psoriasis, keratinocytes provide antimicrobial peptides, such as S100A7 and LL-37, that bind to host DNA and form DNA-LL-37 complexes, which stimulate dendritic cells to produce IFN-alpha and activate myeloid dendritic cells. Activated dendritic cells produce mediators, including IL-12 and IL-23, that lead to T-cells differentiation into Type 1 [35] and Type 17 T-helper cells. Th17 cells play an important role in epithelial immune surveillance [109]. A special focus was set on key pathogenic factors of psoriasis, such as TNF-α, IL-6, IL-8, IL-17, IL-22, IL-23, and IFN-gamma, providing insightful information about disease mechanisms. [51,53,61,62,105]. Additionally, 10 papers have specifically addressed [15,17,47,49,50,56,63,68,104] the genetic basis of psoriasis, with extensive genetic testing that identified more than 50 psoriasis susceptibility loci [27,49,56]. The most important one, PSORS1 [88] is located within the major histocompatibility complex (MHC) on chromosome 6p21 and is directly linked to HLA-Cw6-allele. The gene variants of interest modulate immune pathways and processes that contribute to disease susceptibilities, such as antigen presentation, the IL-23/IL-17 axis, and the type I IFN pathway [49]. A distinctive and interesting approach to psoriasis genetics has been addressed by Sonkoly et al. [68] that identified a specific, dysregulated microRNA expression profile in psoriatic skin compared to healthy skin: miR-203 and miR-125b regulate keratinocyte proliferation and differentiation, while miR-21 inhibits T cell apoptosis. Consequently, research areas referring to therapeutical means have shifted from photochemotherapy and classical immunosuppressant therapies to novel targeted therapies in the last twenty years. Of the specific therapeutic options, this analysis identified the increasingly dominant trend in reporting the use of monoclonal antibodies, twenty-six out of forty-five articles referring to treatment options were focused only on the safety and effectiveness of such novel therapies. The significance of this trend is more accurately reflected by work published in the last decade because ten out of seventeen papers published in this timeframe were focused on treatment, out of which eight addressed specifically various monoclonal antibodies.

Even though only six articles focused specifically on disease comorbidities and four on QoL it is important to mention that these aspects have been uniformly addressed over time and mentioned in other papers, suggesting a constant focus of the research community on these topics. Treatment and management of psoriasis should not address only cutaneous manifestations, but also associated comorbidities and should aim to increase the QoL [117]. Biologics represent a cornerstone in the management of this disease because apart from alleviating skin lesions they seem to work up to a certain extent for associated comorbidities as well. Research focusing on biologics seems to steal the focus in the future as well, for further exploration.

The paper of Fredriksson et al. [106], published in 1978 in *Dermatologica*, was the most cited article in our analysis. It explored the effectiveness of a retinoic acid derivate in treating severe psoriasis. The study was significant at the time because apart from evaluating the effectiveness of a novel retinoid, it was the first article that introduced a currently worldwide used disease severity score, Psoriasis Area Severity Index (PASI). PASI score is currently used in dermatology to assess disease severity and thus, allows the classification of psoriasis in mild, moderate, and severe. Further therapeutic options are selected taking into account various parameters, the PASI score being one of the strongest.

The oldest article included in this analysis, published by Baker et al. [45] focused on generalized pustular psoriasis and identified two etiologically and evolution-wise distinct subtypes of this rare form of psoriasis. The newest one, published by Armstrong et al. [73] offers a state-of-the-art review on clinical presentation, epidemiology, and therapeutic advancements. Due to the fact that the previously mentioned, most recent article included was published in 2020, ongoing research may significantly impact the top 100 articles over the next few years.

This study identified a significant difference in publication and citation patterns in the last two decades compared to before 2000, for which the articles total only 18 and 17.3% of the total citations. This can be explained by the fact that research published before the 2000s focused on pathogenesis, clinics, and conventional treatment options, thus laying the foundation for today’s knowledge about psoriasis. Moreover, these last two decades represent the beginning of immunopathogenesis and biologics.

The collaboration network of authors, countries, and institutions provides an overall picture of the leading researchers in this field on different levels. The United States, Germany, Canada, and the United Kingdom are leaders in the field. Additionally, the most prolific institutions and authors originate in these countries, indicating greater research resources. Moreover, these countries possess some of the most comprehensive and better-updated National Registries, allowing a proper evaluation and follow-up of patients suffering from this disease while also serving as comprehensive research databases. Even though the University of Manchester ranked second, its total link strength is higher than that of the institution ranked first, suggesting a higher connection to other institutions analyzed. The authors that published the most papers were Griffiths, CEM (*n* = 12), Krueger, JG (*n* = 12), Papp, K (*n* = 11), Krueger, GG (*n* = 10), and Menter, A (*n* = 10). On the other hand, when analyzing the authors who contributed to more than five papers based on citations link strength, Nestle, FO, Griffiths, CEM, Papp, K, and Lebwohl, M are proven to be the most influential scholars in their field.

Keywords are a hallmark of the literature, and their analysis can shed light on research and trends in a specific field. The analyzed articles summed up 471 keywords. After setting a minimum threshold of 2 occurrences for each keyword, a bibliometric map based on the 145 eligible items was created and presented in Figure 6. Seven clusters, each defining a research area, were defined. The top 10 keywords with the highest number of occurrences were “quality-of-life”, “skin”, “therapy”, “expression”, “dendritic cells”, “double-blind”, “efficacy”, “necrosis-factor-alpha”, “rheumatoid arthritis”, and “safety”. Figure 7 illustrates the main areas of interest based on the density of keywords in the analyzed articles, where we can observe that the research focuses on quality of life, immune pathways, and treatment safety. Reference co-citation analysis can reflect a domain’s knowledge structure and indicate research hotspots. The analysis showed that the most co-cited references were the papers of Rapp, SR “Psoriasis causes as much disability as other major medical diseases” [89] and Fredrickson, T “Severe psoriasis—oral therapy with a new retinoid” [106], both co-cited 19 times and serving as an additional indicator that treatment and life-quality are main topics in the research field. The works of Rapp, SR, Fredriksson, T, Gelfand, JM, Reich, K, Schon, MP, and Lee, E which bring attention to topics such as pathogenesis and novel treatment options, have the highest link strengths and are located among different clusters, indicating that they may serve as landmarks in the field.

The limitation of this bibliometric analysis resides mainly in the fact that only full-length English articles indexed in the WoS database have been taken into account. This has been partially addressed by not limiting article access type in any kind. Moreover, no time limit has been set when researching articles to be included in the analysis, thus a larger and more accurate overview of the research field has been obtained. To diminish the effect of time on accumulated citations, a citation rate was also calculated in order to identify articles that received a large number of citations over a short period of time. Due to the fact that bibliometric analysis covers a broad area of research, it should be taken into account that papers with the highest citations might address general topics. In order to limit this and to provide an overview of the past, present, and future of psoriasis research, we used a combination of techniques: co-citation analysis to uncover knowledge foundations, bibliographic coupling to understand the present development of themes, and co-word analysis to assess existing or future relationships among topics in psoriasis research.

## 5. Conclusions

Research interest in the scientific community regarding psoriasis is high, leading to the rapid and constant development of this field. This is the first bibliometric study focusing on psoriasis, providing an overview of the intellectual structure and scientific directions in the field, taking into account algorithms based on the top 100 most cited articles on the subject. The research focus is shifting from disease presentation. Treatment modalities, especially novel-targeted therapies, immune pathways, and an integrative, complex approach to such cases are receiving great interest and represent research hotspots in the future.

## Figures and Tables

**Table 1:** Top 100 articles ranked by citation count [15,16,17,18,19,20,21,22,23,24,25,26,27,28,29,30,31,32,33,34,35,36,37,38,39,40,41,42,43,44,45,46,47,48,49,50,51,52,53,54,55,56,57,58,59,60,61,62,63,64,65,66,67,68,69,70,71,72,73,74,75,76,77,78,79,80,81,82,83,84,85,86,87,88,89,90,91,92,93,94,95,96,97,98,99,100,101,102,103,104,105,106,107,108,109,110,111,112,113,114].

Rank	Authors	Journal	Publication Year	Total Citations	Citation Rate
1	Fredriksson et al. [106]	*Dermatologica*	1978	2145	49
2	Nestle et al. [66]	*New England Journal of Medicine*	2009	2041	157
3	Parisi et al. [48]	*Journal of Investigative Dermatology*	2013	1507	167
4	Zheng et al. [62]	*Nature*	2007	1450	97
5	Boehncke et al. [81]	*Lancet*	2015	1338	191
6	Langley et al. [103]	*New England Journal of Medicine*	2014	1337	167
7	Gelfand et al. [102]	*JAMA—Journal of The American Medical Association*	2006	1337	84
8	Van der Fits et al. [54]	*Journal of Immunology*	2009	1290	99
9	Lowes et al. [72]	*Nature*	2007	1291	86
10	Griffiths et al. [85]	*Lancet*	2007	1244	83
11	Leonardi et al. [41]	*Lancet*	2008	1237	88
12	Parrish et al. [77]	*New England Journal of Medicine*	1974	1296	27
13	Papp et al. [40]	*Lancet*	2008	1105	79
14	Rapp et al. [89]	*Journal of The American Academy of Dermatology*	1999	1078	47
15	Nair et al. [47]	*Nature Genetics*	2009	1010	78
16	Lowes et al. [56]	*Annual Review of Immunology,* Vol 3	2014	918	115
17	Leonardi et al. [44]	*New England Journal of Medicine*	2003	934	49
18	Schon et al. [67]	*New England Journal of Medicine*	2005	877	52
19	Reich et al. [60]	*Lancet*	2005	847	50
20	Cargill et al. [17]	*American Journal of Human Genetics*	2007	843	56
21	Nestle et al. [78]	*Journal of Experimental Medicine*	2005	812	48
22	Tyring et al. [42]	*Lancet*	2006	825	52
23	Lowes et al. [96]	*Journal of Investigative Dermatology*	2008	776	55
24	Papp et al. [25]	*New England Journal of Medicine*	2012	743	74
25	Krueger et al. [110]	*Archives of Dermatology*	2001	786	37
26	Leonardi et al. [22]	*New England Journal of Medicine*	2012	733	73
27	Di Cesare et al. [109]	*Journal of Investigative Dermatology*	2009	780	60
28	Wolk et al. [52]	*European Journal of Immunology*	2006	727	45
29	Neimann et al. [80]	*Journal of The American Academy of Dermatology*	2006	739	46
30	Strange et al. [15]	*Nature Genetics*	2010	747	62
31	Lee et al. [57]	*Journal of Experimental Medicine*	2004	689	38
32	Chaudhari et al. [39]	*Lancet*	2001	724	34
33	Menter et al. [21]	*Journal of The American Academy of Dermatology*	2008	677	48
34	Krueger et al. [18]	*New England Journal of Medicine*	2007	617	41
35	Tsoi et al. [49]	*Nature Genetics*	2012	661	66
36	Henseler et al. [92]	*Journal of The American Academy of Dermatology*	1985	660	18
37	Christophers [86]	*Clinical And Experimental Dermatology*	2001	623	30
38	Griffiths et al. [30]	*New England Journal of Medicine*	2010	634	53
39	Lin et al. [65]	*Journal of Immunology*	2011	627	57
40	Marrakchi et al. [63]	*New England Journal of Medicine*	2011	610	55
41	Nograles et al. [108]	*British Journal of Dermatology*	2008	611	44
42	Langley et al. [98]	*Annals of The Rheumatic Diseases*	2005	607	36
43	Arican et al. [105]	*Mediators of Inflammation*	2005	594	35
44	Sonkoly et al. [68]	*Plos One*	2007	577	38
45	Detmar et al. [71]	*Journal of Experimental Medicine*	1994	584	21
46	Griffiths et al. [29]	*Lancet*	2015	592	85
47	Sano et al. [107]	*Nature Medicine*	2005	544	32
48	Papp et al. [16]	*British Journal of Dermatology*	2005	573	34
49	Mcinnes et al. [38]	*Lancet*	2013	561	62
50	Hammarstrom et al. [55]	*Proceedings of The National Academ*	1975	581	12
51	Mrowietz et al. [33]	*Archives of Dermatological Research*	2011	550	50
52	Stern et al. [91]	*Journal of Investigative Dermatology*	2004	558	31
53	Saurat et al. [43]	*British Journal of Dermatology*	2008	550	39
54	Lebwohl et al. [75]	*New England Journal of Medicine*	2015	539	77
55	Rendon et al. [94]	*International Journal of Molecular Sciences*	2019	520	173
56	Chan et al. [53]	*Journal of Experimental Medicine*	2006	510	32
57	Stern et al. [101]	*New England Journal of Medicine*	1979	544	13
58	Rachakonda et al. [95]	*Journal of The American Academy of Dermatology*	2014	535	67
59	Lebwohl [84]	*Lancet*	2003	498	26
60	Gordon et al. [76]	*New England Journal of Medicine*	2016	517	86
61	Gottlieb et al. [100]	*Nature Medicine*	1995	507	19
62	Hollox et al. [90]	*Nature Genetics*	2008	508	36
63	Michalek et al. [20]	*Journal of The European Academy of Dermatology and Venereology*	2017	481	96
64	Pathirana et al. [46]	*Journal of The European Academy of Dermatology and Venereology*	2009	507	39
65	Henseler et al. [34]	*Journal of The American Academy of Dermatology*	1995	500	19
66	Wada et al. [24]	*Plos One*	2012	492	49
67	Ellis et al. [32]	*JAMA—Journal of The American Medical Association*	1986	503	14
68	Kagami et al. [27]	*Journal of Investigative Dermatology*	2010	462	39
69	Blauvelt et al. [36]	*Journal of The American Academy of*	2017	493	99
70	Takeshita et al. [87]	*Journal of The American Academy of*	2017	472	94
71	Nickoloff et al. [99]	*Journal of Clinical Investigation*	2004	415	23
72	Davidovici et al. [88]	*Journal of Investigative Dermatology*	2010	477	40
73	Sommer et al. [58]	*Archives of Dermatological Research*	2006	477	30
74	Abrams et al. [31]	*Journal of Clinical Investigation*	1999	469	20
75	Sugiyama et al. [35]	*Journal of Immunology*	2005	444	26
76	Ma et al. [51]	*Journal of Clinical Investigation*	2008	451	32
77	Stern et al. [64]	*New England Journal of Medicine*	1997	487	19
78	Mehta et al. [74]	*European Heart Journal*	2010	488	41
79	Ellis et al. [113]	*New England Journal of Medicine*	2001	461	22
80	Melski et al. [70]	*Journal of Investigative Dermatology*	1977	483	11
81	Gisondi et al. [82]	*British Journal of Dermatology*	2007	468	31
82	Kurd et al. [112]	*Archives of Dermatology*	2010	474	40
83	Zenz et al. [97]	*Nature*	2005	442	26
84	Hawkes et al. [93]	*Journal of Allergy And Clinical Immunology*	2017	441	88
85	Gelfand et al. [79]	*Archives of Dermatology*	2005	446	26
86	Krueger et al. [111]	*Journal of The American Academy of Dermatology*	2002	431	22
87	Lebwohl et al. [19]	*New England Journal of Medicine*	2003	449	24
88	Naldi et al. [26]	*Journal of Investigative Dermatology*	2005	457	27
89	Gottlieb et al. [59]	*Journal of The American Academy of Dermatology*	2004	439	24
90	Farber et al. [69]	*Dermatologica*	1974	457	10
91	Homey et al. [114]	*Journal of Immunology*	2000	421	19
92	Chiricozzi et al. [61]	*Journal of Investigative Dermatology*	2011	437	40
93	Greb et al. [83]	*Nature Reviews Disease Primers*	2016	424	71
94	Armstrong et al. [73]	*JAMA-Journal of The American Medical Association*	2020	421	211
95	Reich et al. [37]	*Journal of The American Academy of Dermatology*	2017	420	84
96	Nair et al. [104]	*American Journal of Human Genetics*	2006	418	26
97	Trembath et al. [50]	*Human Molecular Genetics*	1997	410	16
98	Gordon et al. [28]	*Journal of The American Academy of Dermatology*	2006	405	25
99	Baker et al. [45]	*British Journal of Dermatology*	1968	420	8
100	Nickoloff et al. [23]	*American Journal of Pathology*	1991	406	13

**Table 2:** Most cited authors.

Rank	Author	Articles	Total Citations
1	Griffiths, CEM	12	9646
2	Krueger, JG	12	7669
3	Papp, K	11	8944
4	Krueger, GG	10	7492
5	Menter, A	10	6945
6	Langley, RG	8	5747
7	Lebwohl, M	8	6318
8	Nestle, F	8	6779
9	Gottlieb, A	7	4080
10	Reich, K	7	5026

**Table 3:** Journal metrics.

Rank	Journal	Total Articles	Total Citations	2021 Impact Factor	5-Year Impact Factor	Average Citations/Publication
1	*New England Journal of Medicine*	16	12,817	176.08	125.16	801.06
2	*Journal of The American Academy of Dermatology*	12	6847	15.48	12.07	570.58
3	*Lancet*	10	8966	202.73	130.84	896.6
4	*Journal of Investigative Dermatology*	8	5377	7.59	8.38	672.13
5	*British Journal of Dermatology*	5	2623	11.11	9.29	524.6
6	*Nature Genetics*	4	2926	41.37	39.32	731.5
7	*Journal of Immunology*	4	2782	5.43	6.17	695.5
8	*Journal of Experimental Medicine*	4	2595	17.57	16.42	648.75
9	*Nature*	3	3183	69.5	63.58	1061
10	*JAMA—Journal of The American Medical Association*	3	2257	157.37	101.12	752.33
11	*Archives of Dermatology*	3	1706	4.78	4.45	568.67
12	*Journal of Clinical Investigation*	3	1335	19.47	19.23	445
13	*Dermatologica*	2	2602	N/A	N/A	1301
14	*American Journal of Human Genetics*	2	1260	11.04	12.87	630
15	*Plos One*	2	1069	3.75	4.06	534.5
16	*Nature Medicine*	2	1051	87.24	68.31	525.5
17	*Archives of Dermatological Research*	2	1025	3.03	3.19	512.5
18	*Journal of The European Academy of Dermatology and Venereology*	2	987	9.22	7.72	493.5
19	*Annual Review of Immunology,* Vol 32	1	917	32.48	35.19	917
20	*European Journal of Immunology*	1	726	6.68	6.09	726
21	*Clinical and Experimental Dermatology*	1	623	4.48	3.19	623
22	*Annals of The Rheumatic Diseases*	1	607	28	20.69	607
23	*Mediators of Inflammation*	1	594	4.52	5.6	594
24	*Proceedings of The National Academy of Sciences of The United States of*	1	581	12.77	13.45	581
25	*Journal of Investigative Dermatology Symposium Proceedings*	1	557	3.73	2.48	557
26	*International Journal of Molecular Sciences*	1	518	6.2	6.62	518
27	*European Heart Journal*	1	488	35.85	33.03	488
28	*Journal of Allergy and Clinical Immunology*	1	440	14.29	13.76	440
29	*Nature Reviews Disease Primers*	1	424	65.03	83.06	424
30	*Human Molecular Genetics*	1	410	5.12	5.99	410
31	*American Journal of Pathology*	1	406	5.77	5.48	406

**Table 4:** Institutions with the most articles.

Rank	Institution	Country	Articles	Citations	Total Link Strength
1	Rockefeller University	USA	13	8201	7
2	University of Manchester	UK	12	9788	11
3	Probity Medical Research	Canada	11	8223	11
4	University of Michigan	USA	10	5384	7
5	University of Utah	USA	10	7097	10
6	Dalhousie University	Canada	9	6354	9
7	Harvard University	USA	9	6605	8
8	Penn University	USA	9	5473	4
9	Saint Louis University	USA	8	6282	8
10	Baylor University	USA	7	4474	7

**Table 5:** Top 10 most used keywords.

Rank	Keyword	No. of Occurrences	Cluster
1	Quality of life	17	1
2	Skin	15	3
3	Therapy	15	7
4	Expression	14	2
5	Dendritic cells	13	2
6	Double-blind	13	4
7	Efficacy	13	4
8	Necrosis factor alpha	13	5
9	Rheumatoid arthritis	12	1
10	Safety	10	4

## Data Availability

All data presented can be made available upon request.
